# Lenvatinib as an Initial Treatment in Patients with Intermediate-Stage Hepatocellular Carcinoma Beyond Up-To-Seven Criteria and Child–Pugh A Liver Function: A Proof-Of-Concept Study

**DOI:** 10.3390/cancers11081084

**Published:** 2019-07-31

**Authors:** Masatoshi Kudo, Kazuomi Ueshima, Stephan Chan, Tomohiro Minami, Hirokazu Chishina, Tomoko Aoki, Masahiro Takita, Satoru Hagiwara, Yasunori Minami, Hiroshi Ida, Mamoru Takenaka, Toshiharu Sakurai, Tomohiro Watanabe, Masahiro Morita, Chikara Ogawa, Yoshiyuki Wada, Masafumi Ikeda, Hiroshi Ishii, Namiki Izumi, Naoshi Nishida

**Affiliations:** 1 Department of Gastroenterology and Hepatology, Kindai University Faculty of Medicine, Osaka-Sayama 589-8511, Japan; 2 State Key Laboratory of Translation Oncology, Sir YK Pao Centre for Cancer, The Chinese University of Hong Kong, Hong Kong 111-1111, China; 3 Department of Gastroenterology, Takamatsu Red Cross Hospital, Takamatsu 760-0017, Japan; 4 Department of Hepatobiliary and Pancreatic Surgery, National Hospital Organization Kyushu Medical Center, Fukuoka 810-8563, Japan; 5 Department of Hepatobiliary and Pancreatic Oncology, National Cancer Center Hospital East, Kashiwa-shi 277-8577, Japan; 6 Department of Gastroenterology, Cancer Institute Hospital of Japanese Foundation for Cancer Research, Ariake 135-8550, Japan; 7 Clinical Research Center, Chiba Cancer Center, Chiba 260-8717, Japan; 8 Department of Gastroenterology, Musashino Red Cross Hospital, Musashino 180-8610, Japan

**Keywords:** hepatocellular carcinoma, lenvatinib, transcatheter arterial chemoembolisation, intermediate stage, up-to-seven criteria

## Abstract

Although transcatheter arterial chemoembolization (TACE) is the standard of care for intermediate-stage hepatocellular carcinoma (HCC), this is a largely heterogeneous disease that includes a subgroup of patients who do not benefit from TACE. The treatment strategy for this subgroup of patients currently remains an unmet need in clinical practice. Here, we performed a proof-of-concept study that lenvatinib may be a more favorable treatment option over TACE as an initial treatment in intermediate-stage HCC patients with large or multinodular tumours exceeding the up-to-seven criteria. This proof-of-concept study included 642 consecutive patients with HCC initially treated with lenvatinib or conventional TACE (cTACE) between January 2006 and December 2018. Of these patients, 176 who received lenvatinib or cTACE as an initial treatment and met the eligibility criteria (unresectable, beyond the up-to-seven criteria, no prior TACE/systemic therapy, no vascular invasion, no extrahepatic spread and Child–Pugh A liver function) were selected for the study. Propensity score matching was used to adjust for patient demographics. After propensity-score matching, the outcome of 30 patients prospectively treated with lenvatinib (14 in clinical trials, one in an early access program and 15 in real world settings) and 60 patients treated with cTACE as the initial treatment was compared. The change of albumin-bilirubin (ALBI) score from baseline to the end of treatment were −2.61 to −2.61 for 30 patients in the lenvatinib group (*p* = 0.254) and −2.66 to −2.09 in the cTACE group (*p* < 0.01), respectively. The lenvatinib group showed a significantly higher objective response rate (73.3% vs. 33.3%; *p* < 0.001) and significantly longer median progression-free survival than the cTACE group (16.0 vs. 3.0 months; *p* < 0.001). Overall survival was significantly longer in the lenvatinib group than in the cTACE group (37.9 vs. 21.3 months; hazard ratio: 0.48, *p* < 0.01). In patients with large or multinodular intermediate-stage HCC exceeding the up-to-seven criteria with Child–Pugh A liver function, who usually do not benefit from TACE, lenvatinib provides a more favorable outcome than TACE.

## 1. Introduction

Hepatocellular carcinoma (HCC) is the third leading cause of cancer-related deaths worldwide and an important health concern [1,2,3]. Recent statistical data indicate that 781,631 people died from HCC worldwide in 2018 [4]. The Barcelona Clinic Liver Cancer (BCLC) staging system is the most widely used treatment algorithm worldwide [5]. Patients with intermediate-stage HCC (i.e., BCLC stage B) are recommended to undergo transcatheter arterial chemoembolization (TACE) as the standard of care. However, BCLC stage B is a very heterogeneous disease in terms of tumor burden and liver function status; therefore, not all patients with intermediate-stage HCC benefit from TACE [6,7]. In order to simplify this heterogeneity, several attempts have been made to subclassify the intermediate-stage HCC to establish a treatment strategy for each substage. The Kinki criteria, one such subclassification, classifies BCLC stage B HCC into the substages B1, B2, and B3 according to the Child–Pugh classification (5–7 points or 8–9 points) combined with the “beyond Milan” criteria and the “within” and “out of” the “up-to-seven” criteria [8,9]. TACE is not effective for substage B2 (up-to-seven out) HCC and also impairs the hepatic functional reserve [10], resulting in poor prognosis. Therefore, TACE is known to be an unsuitable treatment for patients with substage B2 HCC [9]. Treatment strategy of this subgroup of patients is thus still the biggest unmet need in clinical practice worldwide.

Lenvatinib has recently become available as a new molecular targeted agent for the first-line treatment of unresectable HCC in Japan, the USA, the EU, and Asia. Lenvatinib is a multikinase inhibitor that targets vascular endothelial growth factor (VEGF) receptors 1–3, fibroblast growth factor (FGF) receptors 1–4, platelet-derived growth factor (PDGF) receptor alpha, rearranged during transfection (RET), and KIT [11,12,13,14]. The REFLECT trial met its primary endpoint of overall survival (OS) noninferiority of lenvatinib compared with sorafenib, and showed statistically significant and clinically meaningful improvement in the secondary endpoints of progression-free survival (PFS), time to progression, and objective response rate (ORR) [15] in unresectable HCC.

In patients with intermediate-stage HCC, who had prior TACE history and became TACE failures, lenvatinib showed significantly and clinically meaningful anti-cancer efficacy by reducing tumor size or enhancement on dynamic CT in a large proportion of patients (ORR = 61.3%) [9,16,17,18]. In addition, systemic therapy has an advantage over TACE by preserving liver function during the treatment [19,20,21]. Based on this evidence, it was hypothesized that an initial treatment with lenvatinib in patients with intermediate-stage HCC beyond up-to-seven criteria (i.e., B2 sub-stage of HCC), who are not good candidates for TACE and are a subgroup that easily develops TACE refractoriness [22], may provide better outcomes than TACE. Up to now, there has been no report on the efficacy of initial treatment with lenvatinib for intermediate-stage HCC without prior TACE since it is not recommended by worldwide guidelines such as the European Association for the Study of the Liver (EASL) [5], American Association for the Study of Liver Diseases (AASLD) [23], Asian Pacific Association for the Study of the Liver (APASL) [24] or Japan Society of Hepatology (JSH) [25]. The present study aimed to show a proof-of-concept that initial treatment with lenvatinib provides better OS over conventional TACE (cTACE) in BCLC intermediate-stage patients with large or bi-lobar multifocal HCC beyond up-to-seven criteria (i.e., sub-stage B2). This hypothesis/concept was generated based on the fact that lenvatinib showed a high tumor response rate (40.2%) with liver function being maintained during the treatment course for high tumor burden HCC [15].

## 2. Methods

### 2.1. Patients

Between January 2006 and December 2018, lenvatinib treatment was started as an initial treatment in a total of 37 patients with intermediate-stage HCC beyond up-to-seven criteria and Child–Pugh A liver function at multiple centers including Kindai University Hospital, Takamatsu Red Cross Hospital, National Hospital Organization Kyushu Medical Center, National Cancer Center Hospital East, Cancer Institute Hospital of Japanese Foundation for Cancer Research, Chiba Cancer Center, and Musashino Red Cross Hospital. The records of 605 consecutive patients who received cTACE during the same period (2006–2018) were examined, and clinical data obtained at the start, during and the end of cTACE treatment were compared with lenvatinib.

**Figure 1:**
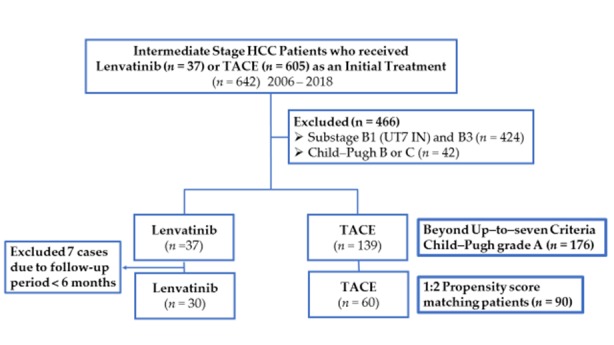
Patients enrolled in this study. A total of 642 patients received lenvatinib or TACE as an initial treatment for intermediate stage HCC between 2006 and 2018. Of them, 37 lenvatinib-treated patients and 139 TACE-treated patients met the eligibility criteria of this study, which is patients with Child–Pugh A liver function and tumor burden of beyond up-to-seven criteria. After propensity score matching, efficacy was compared between 30 lenvatinib-treated patients and 60 TACE-treated patients. TACE; transcatheter arterial chemoembolisation. UT7; up to seven

Lenvatinib administration was performed in a prospective manner since this is not a standard of care as an initial treatment of intermediate-stage HCC and is not recommended by any clinical practice guidelines. The inclusion criteria for both treatments were as follows: (1) unresectable HCC confirmed histologically or cytologically, or confirmed radiologically based on the AASLD criteria; (2) tumor burden beyond up-to-seven criteria; (3) Child–Pugh class A liver function; and (4) Eastern Cooperative Oncology Group performance status 0. Patients were excluded if they had: (1) Child–Pugh class B or C liver function, (2) macroscopic vascular invasion and/or extrahepatic spread, (3) a treatment history of TACE for B2 substage HCC or (4) a history of any systemic therapy. A total of 466 patients were excluded from this study because they did not meet the eligibility criteria. Among 176 patients, 37 patients treated with lenvatinib and 139 patients treated with TACE were included in this study (Table 1, Figure 1). Seven patients treated with lenvatinib who were followed-up for a period shorter than 6 months were excluded before propensity score matching. After propensity score matching, efficacy outcome and the change of liver function were compared between 30 lenvatinib-treated patients and 60 TACE-treated patients.

Of 30 lenvatinib-treated patients, 15 were treated in prospective clinical trials followed by commercially available lenvatinib after the data cut off (one from the phase II trial [26] of lenvatinib started in 2006 and 13 from the phase III REFLECT trial [15]), one patient was treated in the early access program and 15 patients were prospectively treated by commercially available lenvatinib. The median follow-up period was 23 months. The study conformed to the Declaration of Helsinki. The protocol was approved by the ethics committee of Kindai University Faculty of Medicine (Approval number, 27-136).

### 2.2. Treatment Protocol

Lenvatinib (Lenvima^®^; Eisai Co., Ltd., Tokyo, Japan) was administered orally to patients with unresectable HCC. The lenvatinib dose was determined according to body weight as follows: patients weighing <60 kg received 8 mg lenvatinib once daily, whereas those weighing ≥60 kg initially received 12 mg lenvatinib once daily. According to the guidelines for the administration of lenvatinib, the drug dose was reduced, or the treatment was interrupted in patients who developed grade ≥3 severe adverse events (AEs) or any unacceptable grade 2 drug-related AEs. AEs were assessed using the National Cancer Institute Common Terminology Criteria for Adverse Events, version 4.03 [27]. This was maintained until the symptoms resolved as indicated on the package insert. After progression on lenvatinib, second line treatment including TACE, hepatic arterial infusion chemotherapy (HAIC) [28], sorafenib, regorafenib, or investigational drugs were allowed.

TACE was performed as follows: the right femoral artery was accessed with an 18-gauge Seldinger needle, and a 4-Fr sheath was subsequently inserted. The celiac artery was selectively catheterized using a 4-Fr catheter. A 1.9-Fr microcatheter (Shirabe^®^; Piolax, Yokohama, Japan) was advanced coaxially through the catheter into the common or proper hepatic artery. Digital subtraction angiography was performed to evaluate the feeding vessels of targeted HCCs. The tip of the catheter was selectively placed into feeding segmental or subsegmental arteries using selective hepatic angiography and/or tracking navigation imaging when possible. Chemoembolisation was performed using 60–120 mg miriplatin (Miripla^®^; Sumitomo Dainippon Pharma, Osaka, Japan), 20–50 mg epirubicin (Epirubicin^®^; Nippon Kayaku, Tokyo, Japan), or 50–100 mg cisplatin (IAcall^®^; Nippon Kayaku, Tokyo, Japan) mixed with iodised oil (Lipiodol^®^ Ultra-Fluid; Guerbet, Paris, France) followed by injection of gelatine sponge particles (Gelpart^®^; Nippon Kayaku or Gelfoam^®^; Upjohn, Kalamazoo, MI, USA). The injection volume of the emulsion was determined based on the tumor volume (<8 mL). Drug-eluting-bead TACE or balloon-occluded TACE was not performed in this study. After TACE refractoriness, second line treatments including HAIC, sorafenib, regorafenib or investigational drugs were allowed. We adopted the Japan Society of Hepatology criteria of TACE refractoriness, which was defined as follows; TACE refractoriness was defined as an ineffective response or appearance of numerous new lesions after two consecutive TACE procedures that is evident on response evaluation computed tomography (CT) or magnetic resonance imaging (MRI) after 1–3 months, even after chemotherapeutic agents are changed and/or the feeding artery is reanalyzed according to JSH guidelines [29].

### 2.3. Propensity Score Matching

The propensity score was estimated using a logistic regression model fit with the following ten variables: sex, age, hepatitis B virus surface antigen (HBsAg) positivity, hepatitis C antibody positivity, total bilirubin level, serum albumin level, size of intrahepatic lesions (>30 mm), number of intrahepatic lesions (>5), α-fetoprotein (AFP) level and albumin-bilirubin (ALBI) grade. To create a propensity-matched cohort of patients treated with lenvatinib or cTACE (1:2 match), a nearest neighbor-matching algorithm with a greedy heuristic was used [30].

### 2.4. Evaluation of Treatment Response

Treatment response in both groups was evaluated by dynamic CT in accordance with modified Response Evaluation Criteria in Solid Tumor (mRECIST) [31]. Tumor assessments were performed every 6 weeks.

### 2.5. Efficacy Analysis

OS, changes in the ALBI score at each month and at the end of treatment between groups, PFS, ORR, clinical benefit rate (CBR) and disease control rate (DCR) were determined in the propensity score matching cohort. OS was defined as the time from commencement of lenvatinib or initial cTACE until any cause of death. In surviving patients, the censoring date was defined as the last follow-up date. The ALBI score in both groups was compared every month from the date of initiation of treatment until the end of treatment.

PFS was defined as the period from lenvatinib administration or initial cTACE until the time of radiological progression by mRECIST or any cause of death. In patients without progression or death, the censoring date was defined as the last radiological assessment date.

The ORR, CBR and DCR of patients receiving lenvatinib and cTACE were also assessed using mRECIST.

Changes in ALBI score, PFS, OS, ORR, CBR and DCR were also compared between two groups only in patients with ALBI grade 1 liver function.

### 2.6. Statistical Analysis

Data were expressed as the mean and standard deviation. Statistical analyses were performed using Fisher’s exact test, the Kaplan–Meier method, and the log-rank test. The level of significance was set at *p* < 0.05. All analyses were performed using the SPSS Medical Pack for Windows, version 10.0 (SPSS, Inc., Chicago, IL, USA).

## 3. Results

### 3.1. Patient Characteristics

Before propensity score matching, the study included 176 consecutive patients who fulfilled the eligibility criteria and underwent cTACE (*n* = 139) or received lenvatinib (*n* = 37) as the initial treatment during the clinical course of the intermediate stage disease beyond up-to-seven criteria (Figure 1). The characteristics of the patients in the lenvatinib and cTACE groups are summarized in Table 1.

There were 99 (56.3%) anti-HCV Ab-positive patients, 20 (11.4%) HBsAg-positive patients, and 58 (33.0%) who were negative for both HCV Ab and HBsAg. All patients were classified as Child–Pugh A, BCLC-B, and up-to-seven out tumor burden. None of the patients received prior TACE or systemic therapy.

Patient baseline characteristics were similar between the treatment groups, except for serum AFP level. The lenvatinib group consisted of 30 men and seven women with a median age of 68.6 years; the size of intrahepatic lesions was >30 mm in 26 patients, and the number of intrahepatic lesions was >5 in 17 patients. The cTACE group consisted of 106 men and 33 women with a median age of 71.9 years; the size of intrahepatic lesions was >30 mm in 81 patients, and the number of intrahepatic lesions was >5 in 70 patients. The median baseline serum AFP was 101 ng/mL in the lenvatinib group and 28 ng/mL in the cTACE group. Baseline AFP levels <200 ng/mL were more frequent in the cTACE group than in the lenvatinib group (69.8 vs. 48.6%, *p* = 0.02).

After propensity score matching, patient baseline characteristics including serum AFP level were similar between the two groups (Table 1).

### 3.2. Change of Liver Function

**Figure 2:**
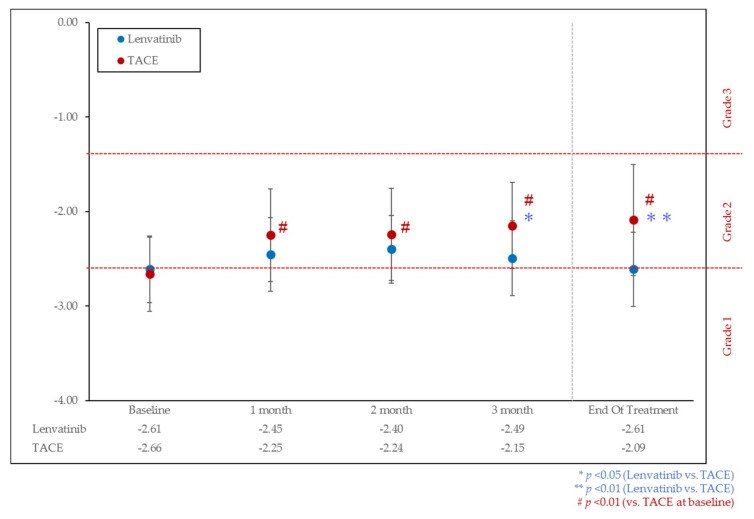
Albumin-bilirubin (ALBI) score over time in lenvatinib and TACE treated groups. ALBI score was significantly worsened at the end of treatment (−2.09) as compared with that at the baseline (−2.66) in the TACE treated group (*p* < 0.01). In contrast, ALBI score was maintained at the baseline (−2.61) and at the end of treatment (−2.61) in the lenvatinib-treated group.

The change of ALBI score from baseline to the end of treatment was −2.61 to −2.61 in 30 patients in the lenvatinib group (*p* = 0.254) and −2.66 to −2.09 in the cTACE group (*p* < 0.01), respectively (Figure 2). In 85.0% of patients in cTACE group, the ALBI score dropped from baseline during the first month.

The ALBI score worsened significantly in the TACE group at each month, especially in the third month and at the end of treatment compared with that in the lenvatinib group (*p* < 0.01, Figure 2).

**Figure A1:**
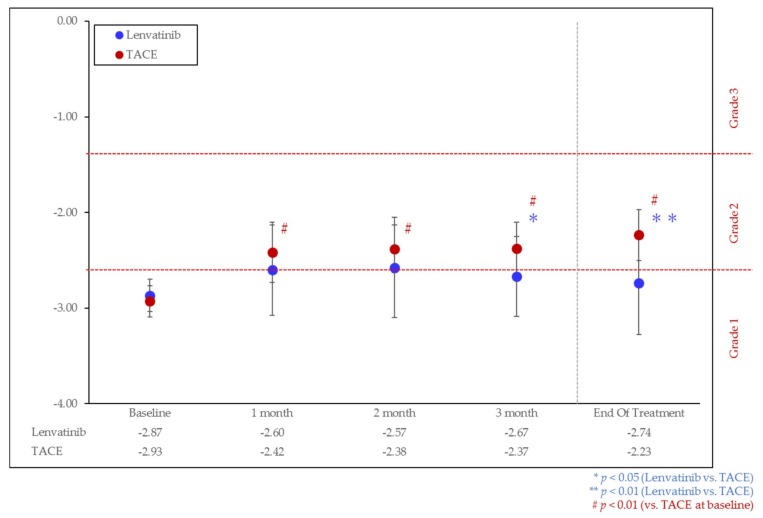
Albumin-bilirubin (ALBI) score over time in patients with ALBI grade 1 treated by lenvatinib (*n* = 19) or TACE (*n* = 37) (propensity score matching results). ALBI score was significantly worsened at the end of treatment (−2.23) as compared with that at the baseline (−2.93) in TACE treated group (*p* < 0.01). In contrast, ALBI score was maintained at the baseline (−2.87) and at the end of treatment (−2.74) in the lenvatinib-treated group.

When confined to patients with ALBI grade 1 liver function, the change of ALBI score from baseline to the end of treatment was −2.87 to −2.74 in 19 lenvatinib-treated patients (*p* = 0.09) and −2.93 to −2.23 in the cTACE group (*p* < 0.01), respectively (Appendix Figure A1).

### 3.3. Efficacy and Safety

**Figure 3:**
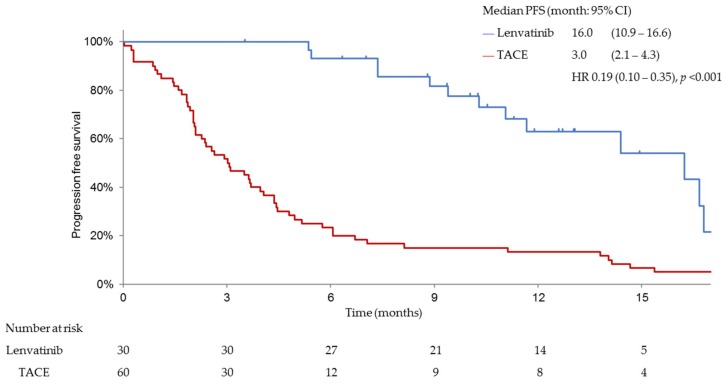
Progression free survival (PFS) in both groups with Child–Pugh A liver function after propensity score matching. PFS in the lenvatinib-treated group was significantly better than that in the TACE-treated group (16.0 months vs. 3.0 months; hazard ratio (HR) 0.19, *p* < 0.001).

**Figure 4:**
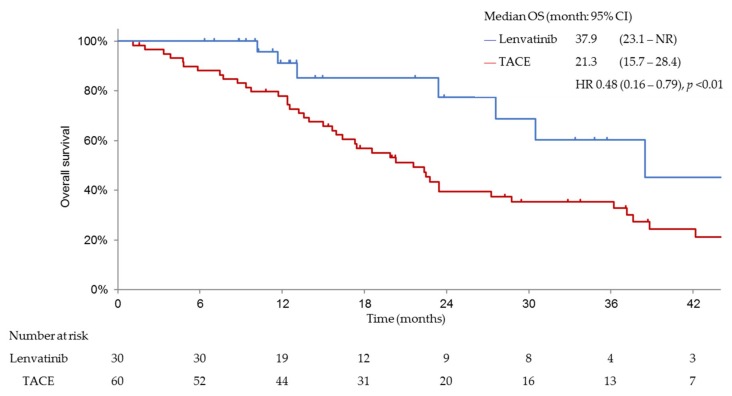
Overall survival (OS) in both groups after propensity score matching. OS in the lenvatinib-treated group was significantly better than that in the TACE-treated group (37.9 months vs. 21.3 months; hazard ratio (HR)0.48, *p* < 0.01). NR; not reached

PFS, ORR, CBR and DCR were significantly better in patients treated with lenvatinib than in those treated with cTACE. The median PFS was 16.0 months (95% confidence interval (CI), 10.9–16.6) for patients in the lenvatinib group and 3.0 months (95% CI, 2.1–4.3) for patients in the cTACE group (hazard ratio (HR), 0.19; 95% CI, 0.10–0.35; *p* < 0.001; Figure 3). Patients treated with lenvatinib showed significantly better ORR (complete response (CR) plus partial response (PR)) than those receiving cTACE as per mRECIST (73.3% vs. 33.3%; odds ratio, 0.18 (95% CI, 0.07–0.48; *p* < 0.001)) (Table 2). The median OS was significantly longer in patients with intermediate-stage beyond up-to-seven HCC and baseline Child–Pugh class A, who received lenvatinib (*n* = 30) (37.9 months (95% CI, 23.1–NR)) than in those who underwent cTACE (*n* = 60) (21.3 months (95% CI, 15.7–28.4); HR, 0.48; 95% CI, 0.16–0.79; *p* < 0.01; Figure 4).

The median treatment duration in the lenvatinib group was 13.1 months, whereas that in the repeated TACE group was 8.2 months (median TACE cycles: three). There was no lenvatinib discontinuation patients due to adverse events. Four patients achieved drug free after CR in the lenvatinib-treated group; CR with drug free was obtained in one patient by lenvatinib alone and in three patients by lenvatinib followed by additional selective cTACE to the remaining viable tumor during the ongoing response with lenvatinib (lenvatinib-TACE sequential therapy). In 14 out of 30 lenvatinib-treated patients, lenvatinib is still ongoing because of continuing response. After treatment discontinuation of lenvatinib either due to ongoing response including CR (*n* = 2) or progression (*n* = 1), patients received conversion therapy such as TACE or second line therapy with sorafenib, HAIC, investigational drugs, ablation or resection. Because of the high response rate of lenvatinib, two patients achieved down staging and could have received ablation (*n* = 1) or resection (*n* = 1). This is not the case in cTACE group. In 10 of 16 (62.5%) patients who discontinued lenvatinib during sustained response or after progression and received cTACE, three achieved CR and seven achieved PR with additional TACE. In the initial TACE-treated group, HAIC, sorafenib or clinical trials with investigational drugs were performed after TACE refractoriness.

The mean dose intensities in the lenvatinib group were 6.3 mg/day and 9.8 mg/day for the groups with starting doses of 8 mg and 12 mg, respectively. The median time to first dose reduction was 29.9 weeks for lenvatinib. Eleven patients (52.4%) maintained the starting dose of 8 mg or 12 mg. In the cTACE group, the median number of TACE procedures was three.

**Figure A2:**
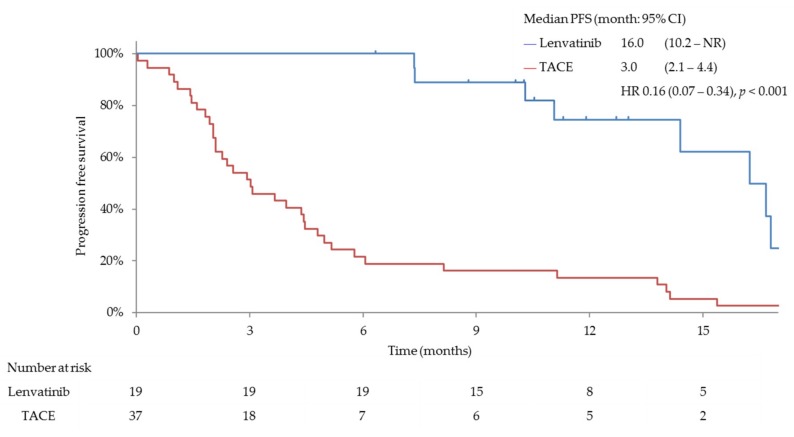
Progression free survival (PFS) in both groups with ALBI grade 1 liver function after propensity score matching. PFS in the lenvatinib-treated group was significantly better than that in the TACE-treated group (16.0 months vs. 3.0 months; HR 0.16, *p* < 0.001). NR; not reached.

**Figure A3:**
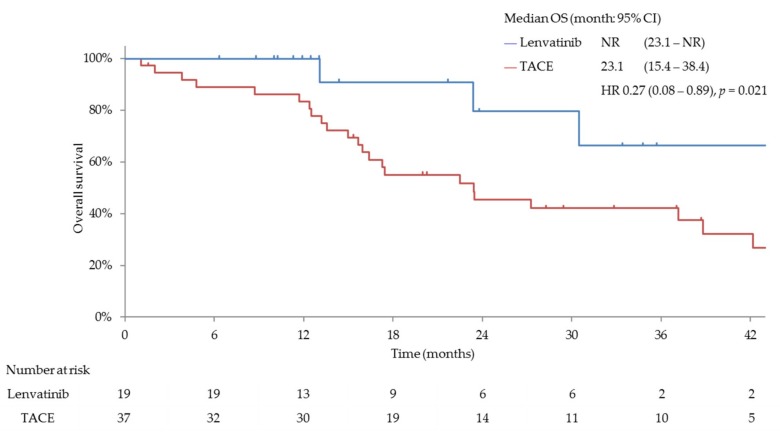
Overall survival (OS) in both groups with ALBI grade 1 after propensity score matching. OS in the lenvatinib-treated group was significantly better than that in the TACE-treated group (not reached (NR) vs. 23.1 months; HR 0.27, *p* = 0.021). NR; not reached.

When confined to patients with ALBI grade 1 liver function, lenvatinib-treated patients showed better results than TACE-treated patients in terms of ORR (73.7% vs. 37.8%, odds ratio; 4.47, *p* < 0.05), PFS (16.0 months vs. 3.0 months, HR 0.16, *p* < 0.001) and OS (not reached vs. 23.1 months, HR 0.27, *p* = 0.021) as compared with the results obtained in patients with Child–Pugh A liver function (Appendix Figure A2 and Appendix Figure A3 and Appendix Table A1).

Regarding the safety of lenvatinib treatment, no severe AEs or no new safety signals were observed in the 30 lenvatinib-treated patients.

## 4. Discussion

To the best of our knowledge, the present study is the first to demonstrate the efficacy of initial therapy with a molecular targeted agent, lenvatinib, on the OS in patients with intermediate-stage HCC beyond up-to-seven criteria and Child–Pugh A liver function as compared with that in TACE-treated patients. Propensity-score matching analysis was used to address the potential bias associated with differences in patient background. The results showed that lenvatinib is superior to TACE, the current standard of care in intermediate-stage HCC, as an initial treatment for patients with large or bi-lobar multifocal intermediate-stage (beyond up-to-seven criteria) HCC in terms of ORR, PFS, CBR, DCR and OS. Lenvatinib was also associated with better preservation of liver function than cTACE both during and after the treatment course. This study provides proof-of-concept that an effective systemic agent is a potentially better initial treatment than cTACE in large or multifocal bilobar HCC.

One of the key points in HCC treatment is to preserve liver function as much as possible in addition to achieving a high tumor response. Several trials reported acute and chronic liver function deterioration were observed in patients treated with TACE, especially in those receiving less selective TACE procedures for large tumor burdens [10,32,33]. Liver function impairment is reportedly detected earlier in BCLC stage B patients who do not meet the up-to-seven criteria (B2 substage) than in those who meet the up-to-seven criteria (B1 substage) [10,32]. In B2 substage HCC, the early onset of TACE refractoriness is responsible for the shorter survival than that of patients with B1 substage HCC [10,32]. In another retrospective study [34], multivariate analysis revealed that beyond the up-to-seven criteria is an independent factor associated with Child–Pugh class deterioration (HR, 1.9; *p* = 0.005). These findings are consistent with the results of the present study, as TACE led to the deterioration of liver function in patients with bi-lobar multifocal intermediate-stage HCC. By contrast, lenvatinib was associated with the maintenance of liver function in patients treated with lenvatinib than in those undergoing TACE. These data support the use of lenvatinib as the initial treatment to prevent liver function deterioration in TACE-unsuitable patients with a tumor burden beyond up-to-seven criteria. The OS in TACE-treated intermediate stage HCC patients beyond up-to-seven criteria was reportedly 20.4–27.6 months in large Japanese cohorts [35,36], consistent with the OS initially treated with TACE in the present study. In contrast, OS in patients who received initial treatment with lenvatinib followed or not followed by additional TACE in the present study showed a better survival of 37.9 months.

In the present study, the ORR of patients who received lenvatinib therapy was considerably higher (ORR = 73.3%) than that reported in the REFLECT trial (40.6%); however, the data were consistent with the results of a Japanese subpopulation analysis (ORR = 61.3%) [16] in intermediate-stage HCC patients with Child–Pugh A liver function, including patients with a history of prior TACE. Similarly, subgroup analysis in the REFLECT trial also showed that the ORR was higher in patients with intermediate-stage HCC than in patients with advanced-stage HCC [15]. One possible explanation for the even higher ORR in the present study is that all patients had intermediate-stage HCC and Child–Pugh class A liver function with higher proportion of ALBI grade 1 because of no history of prior TACE in all 30 patients as compared with Japanese subpopulation analysis [16]. Preserved liver function is an important factor for ORR and outcome of HCC patients in systemic therapy [37,38].

Another unfavorable aspect of TACE for high tumor burden HCC is that incomplete TACE increases tumor hypoxia, leading to the upregulation of hypoxia inducible factor-1-α (HIF1-α) [39,40,41]. Increased HIF1-α, in turn, upregulates the expression of VEGF, FGF or PDGF and increases tumor angiogenesis [39,40,41,42]. That is, giving TACE to intermediate-stage HCC patients with up-to-seven criteria out leads to a spike in the intra-tumoral concentration of VEGF, FGF or PDGF, suggesting that blockade of these receptors may prevent the effects of a surge in proangiogenic factors [39,40]. A preclinical model has shown that the combination of antiangiogenic therapy with TACE reduces tumor volume and vessel density, as well as prolonging survival, when compared with TACE alone [43]. This is one possible rationale of pre-treatment with an antiangiogenic agent before TACE, which results in a favorable outcome in high tumor burden HCC. Indeed, in the present study, three lenvatinib-treated patients achieved CR and eventually achieved drug-free status after additional effective cTACE during considerable tumor shrinkage/necrosis compared with baseline on lenvatinib (lenvatinib-TACE sequential therapy).

In the present study, 62.5% of lenvatinib-treated patients received additional TACE during ongoing response or after progression on lenvatinib. However, most of the TACE procedures were performed superselectively, resulting in high objective response and preservation of liver function since remaining tumors after lenvatinib are fewer as compared with the baseline tumor burden. In addition, as is very well known, antiangiogenic agents play a very important role in the normalization of tumor vasculature [44] and in the enhancement of the effective, homogeneous delivery of anticancer agents (including lipiodol and gelatin sponge) into tumor tissues [45], resulting in improved responses to TACE as compared with performing TACE without pretreatment with lenvatinib.

The present results indicated that lenvatinib prolonged OS by preserving liver function and improving PFS and ORR compared with cTACE in patients with up-to-seven criteria out intermediate-stage HCC, a current unmet need. In patients with intermediate-stage HCC within the up-to-seven criteria who are not candidates for resection or ablation, superselective cTACE may remain the standard of care, as superselective cTACE achieves relatively high responses and preserves liver function. However, in patients beyond the up-to-seven criteria, lenvatinib may be the first choice of treatment because it can prevent liver function deterioration and achieve higher response rates than TACE.

The present study had two limitations. First, the number of patients analyzed was relatively small since this is a proof-of-concept study. Second, the retrospective analysis of the study (especially on the analysis of TACE efficacy) may have led to bias in patient selection. This limitation was overcome by propensity score matching, which mitigated the potential confounding selection bias of this non-randomized study. To validate the findings of this proof-of-concept study, a prospective randomized controlled trial would be of value to prove the clinical benefit of lenvatinib as an initial treatment in patients with up-to-seven out intermediate-stage HCC.

## 5. Conclusions

The current proof-of-concept study showed that in patients with large or multinodular intermediate-stage HCC beyond the up-to-seven criteria with Child–Pugh A liver function, lenvatinib is associated with better OS than TACE due to high ORR/CBR/DCR, better PFS and better preservation of liver function. Lenvatinib may be a preferred first-line therapy over cTACE in subpopulation of large and multifocal bi-lobar intermediate-stage HCC. This study confirmed the proof-of-concept that a prospective randomized controlled trial would be worth performing in order to solve the current unmet need and establish the new standard of care for this stage of HCC.

## Figures and Tables

**Table 1:** Patient disease characteristics at the time of study entry before and after propensity score matching.

Characteristics	Before Matching			After Matching		
	Lenvatinib *n* = 37 (%)	TACE *n* = 139 (%)	*p* Value	Lenvatinib *n* = 30 (%)	TACE *n* = 60 (%)	*p* Value
Age, mean	68.6	71.9	0.173	68.2	72.4	0.272
Gender, male	30 (81.1%)	106 (76.3%)	0.661	24 (80.0%)	42 (70.0%)	0.449
HCV positive	15 (40.5%)	84 (60.4%)	0.040	12 (40.0%)	29 (48.3%)	0.506
HBV positive	8 (21.6%)	12 (8.6%)	0.039	7 (23.3%)	10 (16.7%)	0.430
Alcohol abuse	6 (16.2%)	27 (19.4%)	0.814	3 (10.0%)	10 (16.7%)	0.532
Size of intrahepatic lesion, >30 mm	26 (70.3%)	81 (58.3%)	0.255	20 (66.7%)	39 (65.0%)	1.000
Number of intrahepatic lesion, >5	17 (45.9%)	70 (50.4%)	0.713	14 (46.7%)	35 (45.0%)	0.371
EHS positive	0 (0%)	0 (0%)	1.000	0 (0%)	0 (0%)	1.000
MVI positive	0 (0%)	0 (0%)	1.000	0 (0%)	0 (0%)	1.000
Child–Pugh score 5A	25 (67.6%)	91 (65.5%)	0.848	20 (66.7%)	37 (61.7%)	0.817
Child–Pugh score 6A	12 (32.4%)	48 (34.5%)	0.848	10 (33.3%)	23 (38.3%)	0.817
Child–Pugh score ≥ 7	0 (0%)	0 (0%)	1.000	0 (0%)	0 (0%)	1.000
ALBI grade 1	22 (59.5%)	66 (47.5%)	0.267	19 (63.3%)	37 (61.7%)	0.880
Albumin, median (g/dL)	4.0	3.8	0.092	4.0	3.9	0.881
Total bilirubin, median (mg/dL)	0.7	0.7	0.098	0.7	0.7	0.293
Baseline AFP ≥ 200 ng/mL	18 (48.6%)	97 (69.8%)	0.020	15 (50.0%)	28 (46.7%)	0.825
Baseline AFP, median (ng/mL)	101	28	0.049	103	107	0.355

TACE: transcatheter arterial chemoembolization; HCV: hepatitis C virus; HBV: hepatitis B virus; EHS: extrahepatic spread;MVI: Macrovascular invasion; ALBI grade: albumin-bilirubin grade; AFP; α-fetoprotein concentration.

**Table 2:** Objective response rate (ORR) (propensity score matching).

Response Category	Lenvatinib *n* = 30 (%)	TACE *n* = 60 (%)	*p*-Value Odds Ratio (95% CI)
ORR	22 (73.3%)	20 (33.3%)	*p* < 0.001
			5.39 (1.90–16.67)
CBR(CR + PR + SD ≥ 24w)	29 (96.7%)	22 (36.7%)	*p* < 0.001
			48.1 (7.01–2073.85)
DCR	30 (100.0%)	32 (53.3%)	
CR	2	4	
PR	20	16	
SD	8	12	
Durable stable disease (SD ≥ 24w)	7	2	
PD	0	26	
NE	0	2	

CI: confidence interval; ORR: objective response rate; CBR: clinical benefit rate; DCR: disease control rate; CR: complete response; PR: partial response; SD: stable disease; PD: progressive disease; NE: not evaluable.

## Appendix A

**Table A1:** Objective response rate in both groups with ALBI grade 1 liver function.

Response Category	Lenvatinib *n* = 19 (%)	TACE *n* = 37 (%)	*p*-Value Odds Ratio (95% CI)
ORR	14 (73.7%)	14 (37.8%)	*p* < 0.05
			4.47 (1.19–19.51)
CBR (CR + PR + SD ≥ 24w)	19 (100.0%)	16 (43.2%)	
DCR	19 (100.0%)	22 (59.5%)	
CR	1	4	
PR	13	10	
SD	5	8	
Durable stable disease (SD ≥ 24w)	5	2	
PD	0	13	
NE	0	2	
